# Species turnover in ant assemblages is greater horizontally than vertically in the world's tallest tropical forest

**DOI:** 10.1002/ece3.9158

**Published:** 2022-07-29

**Authors:** Shuang Xing, Amelia S. C. Hood, Roman J. Dial, Tom M. Fayle

**Affiliations:** ^1^ School of Ecology Sun Yat‐Sen University Guangzhou P. R. China; ^2^ Biology Centre of Czech Academy of Sciences Institute of Entomology Ceske Budejovice Czech Republic; ^3^ Department of Zoology University of Cambridge Cambridge UK; ^4^ Centre for Agri‐Environmental Research, School of Agriculture, Policy and Development University of Reading Reading UK; ^5^ Institute of Culture and Environment Alaska Pacific University Anchorage Alaska USA; ^6^ School of Biological and Behavioural Sciences Queen Mary University of London London UK

**Keywords:** community ecology, distance‐decay, habitat complexity, microclimate, species turnover, vertical stratification

## Abstract

Abiotic and biotic factors structure species assembly in ecosystems both horizontally and vertically. However, the way community composition changes along comparable horizontal and vertical distances in complex three‐dimensional habitats, and the factors driving these patterns, remains poorly understood. By sampling ant assemblages at comparable vertical and horizontal spatial scales in a tropical rainforest, we tested hypotheses that predicted differences in vertical and horizontal turnover explained by different drivers in vertical and horizontal space. These drivers included environmental filtering, such as microclimate (temperature, humidity, and photosynthetic photon flux density) and microhabitat connectivity (leaf area), which are structured differently across vertical and horizontal space. We found that both ant abundance and richness decreased significantly with increasing vertical height. Although the dissimilarity between ant assemblages increased with vertical distance, indicating a clear distance‐decay pattern, the dissimilarity was higher horizontally where it appeared independent of distance. The pronounced horizontal and vertical structuring of ant assemblages across short distances is likely explained by a combination of microclimate and microhabitat connectivity. Our results demonstrate the importance of considering three‐dimensional spatial variation in local assemblages and reveal how highly diverse communities can be supported by complex habitats.

## Graphical abstract

By sampling ant assemblages at the comparable vertical and horizontal spatial scales in a tropical rain forest, we detected increasing difference in assemblage composition across vertical forest strata, but no effects of horizontal distance on assemblage turnover across trees. Our research highlights the importance of considering assemblage turnover in both horizontal and vertical dimensions and demonstrates how these distance‐decay patterns are influenced by microclimate and habitat connectivity within a structurally complex habitat.

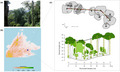

## 1 INTRODUCTION

Vertical stratification has long been recognized as one of the key ecological mechanisms in structuring species distributions and diversity patterns (MacArthur & MacArthur, 1961). Habitats with high vertical complexity, such as forests, often house more species than habitats with low structural complexity (Oliveira & Scheffers, 2019). Vertical stratification of communities has been documented for a range of habitats, from the deep ocean to tropical forest, and is largely driven by variation in abiotic conditions and resources (Jorda et al., 2020; Sheehan et al., 2019; Venegas‐Li et al., 2018). For example, in pelagic ecosystems, temperature changes are one of the main drivers that induce vertical stratification (Li et al., 2020), whereas in terrestrial ecosystems the heterogeneity in vegetation structure across different heights can be important (Jarron et al., 2020).

Although the effects of horizontal distance on species turnover have been examined at large spatial scales (kilometers; Chesters et al., 2019), it is often considered to be negligible at small scales (metres), and therefore has rarely been incorporated into studies on vertical stratification patterns (Roisin et al., 2006; Weiss et al., 2016). However, in complex ecosystems with high three‐dimensional structural heterogeneity, such as coral reefs and tropical rainforests, abiotic and biotic factors can vary greatly both horizontally and vertically across short distances (Reaka‐Kudla, 1997), driving small‐scale variation in community composition (Davies & Asner, 2014). Although numerous studies investigate species diversity changes across horizontal and vertical dimensions, few assess both simultaneously at comparable spatial scales (Wermelinger et al., 2007).

Tropical rainforests are considered the most structurally complex and biologically diverse terrestrial habitat on Earth (Ehrlich & Wilson, 1991) because vegetation complexity, light intensity, temperature, and humidity generate substantial microclimatic and structural variability (Nakamura et al., 2017; Parker, 1995; Scheffers et al., 2013, 2017). With increasing height, air temperature tends to increase while relative humidity tends to decrease with the interaction between solar radiation and canopy buffering (Scheffers et al., 2013). This often leads to the vertical migration of species during transitions between dry and wet seasons (Basham & Scheffers, 2020). Horizontally, microclimate often varies more between open forest gaps and shaded closed canopy forests (Fetcher et al., 1985; Parker, 1995; Scheffers et al., 2017) than between ground and canopy surface. For example, maximum air temperature can vary up to 2.2°C vertically (Scheffers et al., 2013), while differs by 8°C between gaps and closed canopy over similar scales (metres; Brown, 1993; Kaspari et al., 2015; Stark et al., 2017). Finally, microhabitat structure, especially connectivity, can play an important role in shaping community composition in rainforest (Adams et al., 2019; Beaulieu et al., 2010; Ramette & Tiedje, 2007). In many habitats, inter‐arboreal connectivity is low, and this limits horizontal movement for nonflying arboreal organisms (Adams et al., 2017). In the rainforest, connecting physical structures (e.g., lianas) are common, and they facilitate movement, thereby increasing connectivity and decreasing turnover of nonflying arboreal organisms (Adams et al., 2017; Adams et al., 2019; Bélisle, 2005). There are other characteristics that affect faunal turnover in rainforests, e.g., plant composition and nutrient distribution (Vieira & Monteiro‐Filho, 2003), but the influence of these characteristics is likely context specific.

Those studies that do consider community composition both horizontally and vertically in tropical rainforests, find patterns that are idiosyncratic and taxon‐dependent (Antoniazzi et al., 2021; Basham et al., 2018). The relevant scale of turnover can depend on behavioral, morphological, and physiological traits of organisms interacting with habitat variability (Soininen et al., 2018). For amphibians in Madagascar, distance‐decay occurs in the canopy and understory but not on the ground, most simply explained by limited canopy connectivity (Basham et al., 2018). Conversely, for ants in secondary forests in Mexico, distance‐decay occurs in ground assemblages but not in canopy assemblages, which may result from the easier movement by smaller animals combined with larger territories of canopy ants, and higher microhabitat heterogeneity at the ground level (Antoniazzi et al., 2021). Basset et al. (2015) revealed that the composition turnover was structured by vertical, horizontal, and seasonal dimensions but is more similar within the horizontal dimension. Nevertheless, how assemblage turnover within rainforest fauna varies at comparable horizontal and vertical distances remains largely unknown (Dial, Bloodworth, et al., 2004; Nakamura et al., 2017), partly due to the technical challenges in conducting sampling across replicated horizontal positions for a range of vertical heights (Dial, Bloodworth, et al., 2004).

Tropical arboreal ants are abundant and ecologically important, offering an ideal study system for examining spatial turnover in tropical forest canopies (Yusah et al., 2018). The distribution and activity of ants in tropical forests are sensitive to microclimate (Kaspari, 1993; Perfecto & Vandermeer, 1996), which can vary greatly both vertically and horizontally. Key food resources such as carbohydrates and protein for ants change with height in the canopy (Kaspari & Yanoviak, 2001), suggesting rapid ant community turnover vertically. A large‐scale experiment demonstrates that the species richness, composition, and beta diversity of ants change after lianas are removed (Adams et al., 2019), suggesting that high connectivity reduces horizontal turnover.

**FIGURE 1:**
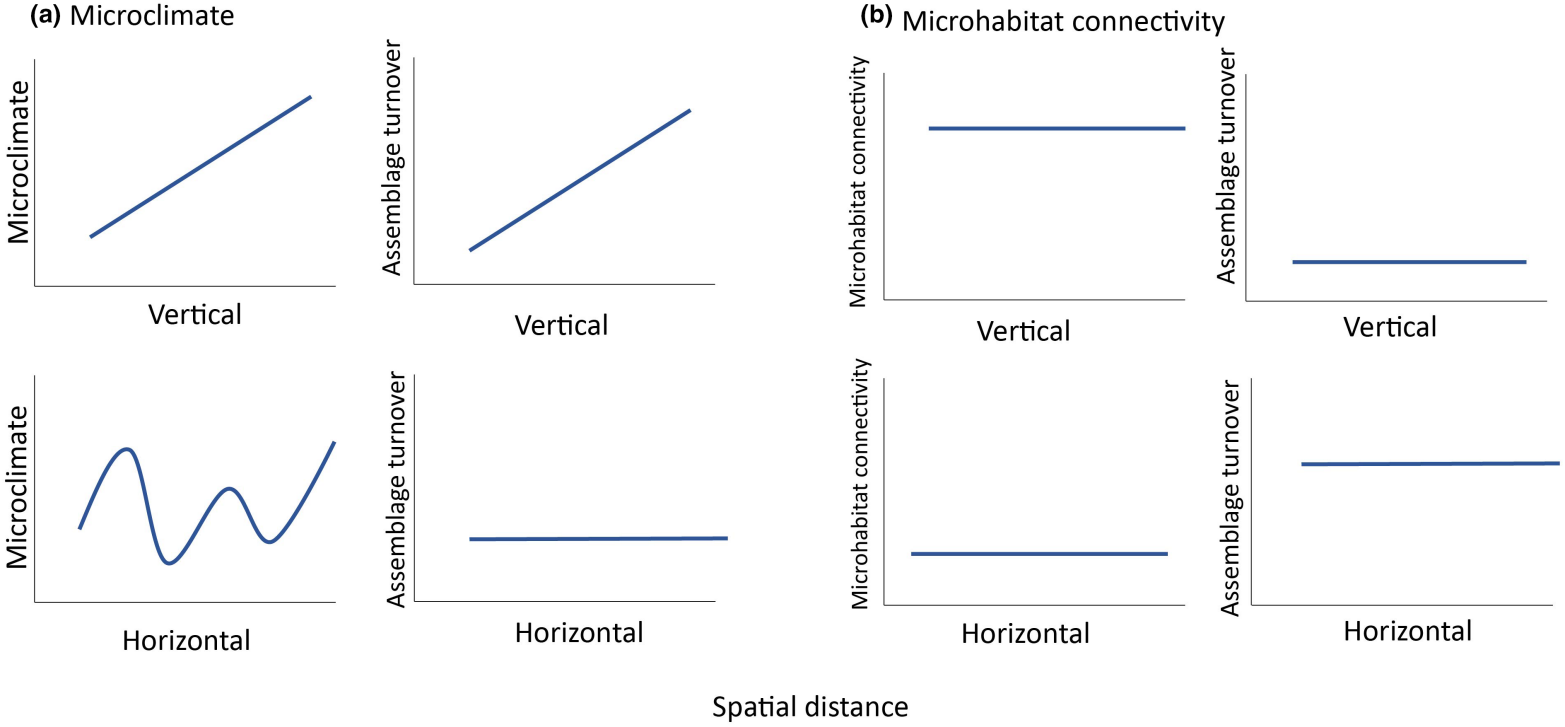
Predictions of how ant diversity could be influenced by microclimate and microhabitat connectivity. (a) The scenario in which microclimate is the dominant driver shaping turnover of ant assemblages across vertical and horizontal dimensions. In the vertical dimension, microclimate tends to change linearly with vertical height, resulting in a linear relationship between vertical distance and assemblage turnover. In contrast, microclimate can vary nonlinearly across horizontal space, leading to weak or no relationship between distance and turnover. (b) The simplified scenario when microhabitat connectivity dominates in shaping turnover across horizontal and vertical dimensions at small scales. Vertically, microhabitat connectivity is high regardless of distance, leaving no effect of vertical distance on turnover. Horizontally, microhabitat connectivity is low across all distances, resulting in weak or no relationship between horizontal distance and turnover. However, lower connectivity horizontally than vertically at comparable distances creates higher horizontal than vertical turnover.

Using canopy traverse techniques (Dial et al., 2006; Dial, Sillett, et al., 2004), we mapped arboreal ant taxa at a small spatial scale from the ground to the canopy across different horizontal positions in a tropical rainforest in Sabah, Malaysia, home to both extremely high arthropod biodiversity and the world's tallest tropical trees (Shenkin et al., 2019). We compared pairwise dissimilarity of ant assemblages across vertical and horizontal distances, microclimate, and microhabitat connectivity. Specifically, we tested two, not mutually exclusive hypotheses (Figure 1):

1. Microclimate will generate a turnover of ant species along environmental gradients (such as light intensity, temperature, and/or humidity) at small scales both vertically and horizontally.
2. Microhabitat connectivity will reduce vertical turnover due to tree architecture (trunks) compared with horizontal turnover.

## 2 MATERIALS AND METHODS

### 2.1 Study area

**FIGURE 2:**
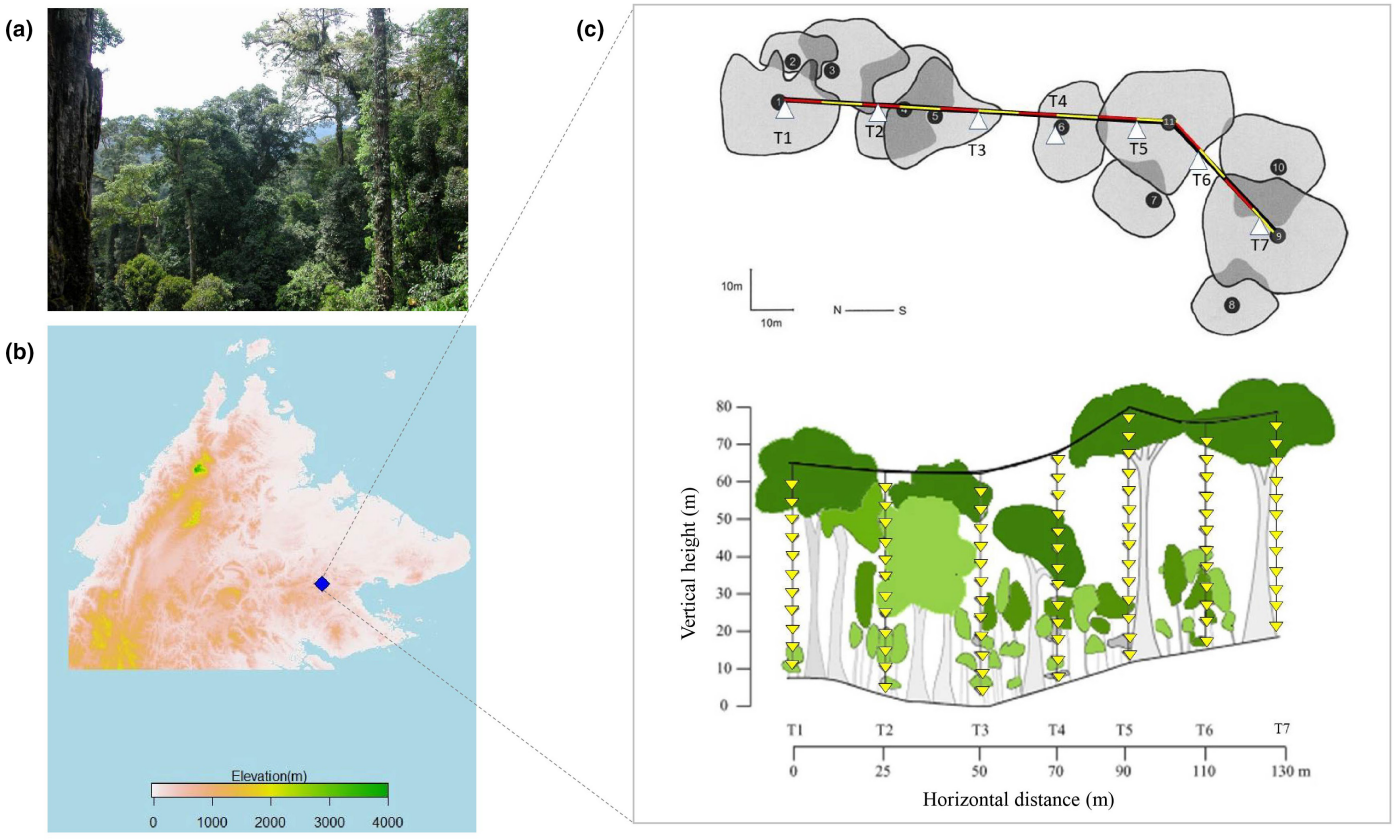
Study site and design: (a) habitat photo of the sampling plot; (b) map of Sabah with elevation gradient presented (metres above sea level); (c) the top and side view of the transect established within the forest plot (modified from Dial, Sillett, et al., 2004; Dial et al., 2006).

We conducted field sampling during April and May 2002 in primary lowland dipterocarp rainforest, part of a Class I forest reserve near Danum Valley Field Centre, Sabah, Malaysian Borneo (4°57′N, 117°48′E; Dial, Sillett, et al., 2004; Dial et al., 2006; Figure 2). The mean temperature is 26.7°C ± 1.9°C, relative humidity ranges from 72% to 100% (saturation) daily, and mean annual rainfall is 2669 mm (range: 1918–3294 mm; Walsh & Newbery, 1999). Because of its equatorial position, the forest is subject to two rainy (May–June and October–January) and two dry seasons (March–April and August–September; Walsh & Newbery, 1999).

Sampling was conducted in a 160 m long × 70 m tall vertical slice of primary forest at 150 m a.s.l., spanning a small tributary stream of the Segama River (Figure 2). The ground was relatively level within the plot, without steep hillsides or ridges (Dial, Sillett, et al., 2004). There were 11 trees from four families with canopies in contact with the transect, with nine individuals in the Dipterocarpaceae and Fabaceae families (Dial et al., 2006). These trees ranged from 40.8 m to 75.0 m in height (see Table 1 for details of tree species) above an overstory of unidentified trees.

**TABLE 1:** The species name and trait information of the 11 trees sampled that comprising the traverse

Key	Species	Family	Height (m)	Crown spread (m)
1	*Shorea leprosula*	Dipterocarpaceae	66.0	28.4
2	*Parashorea malaanonan*	Dipterocarpaceae	55.8	14.0
3	*S. parvifolia*	Dipterocarpaceae	59.2	20.9
4	*Pentaspodon motleyi*	Anacardiaceae	52.6	26.8
5	*S. johorensis*	Dipterocarpaceae	63.0	22.8
6	*S. johorensis*	Dipterocarpaceae	48.0	20.4
7	*Dialium indum*	Fabaceae	40.8	17.4
8	*Azadirachta excelsa*	Meliaceae	61.9	19.0
9	*Parashorea malaanonan*	Dipterocarpaceae	72.8	32.2
10	*P. tomentella*	Dipterocarpaceae	59.2	25.4
11	*Koompassia excelsa*	Fabaceae	75.0	29.7

### 2.2 Field site

The field survey consisted of several activities. First, we established the vertical sampling system in April 2002 via a 200 m long application of “canopy trekking” to access the forest trees to secure a horizontal traverse line (Dial et al., 2006; Dial, Sillett, et al., 2004). Once the 130 m traverse line was secured near the top of the three tallest trees, each >65 m above ground level, we suspended seven vertical transects (Figure 2). We used these vertical transects to sample the canopy interior systematically from 1 m above ground to the traverse line 55–65 m above ground. Finally, we collected measures of photosynthetically active radiation instantaneous with a handheld meter, temperature, and humidity with suspended data logging sensors over 24 h, and fogging samples in May 2002 (Dial et al., 2006).

#### 2.2.1 Quantifying ant assemblages

Ant assemblages were sampled using insecticide fogging between 0700 and 0930 on 12–24 May 2002 from within the canopy by a climber rappelling down using a Swing‐Fog model SN50 (Phoenix Fogger) near each of seven vertical sample transects. These vertical transects were suspended from a 130 m horizontal traverse line secured in the upper canopy and were arranged at 20–25 m intervals horizontally (Figure 2). Each of these transects supported multiple individual circular fogging trays (1 m^2^) (*n* = 86) suspended in the air with attached ethanol‐filled collecting bottles spaced at approximately 5 m vertical intervals beginning at 1 m above the ground (Figure 2). These trays collected knocked‐down arthropods that were between trays at the time of fogging. A 1.6% aqueous solution of the synthetic pyrethrum (Cypermethrin) was used. Arthropods were collected into 80% ethanol 1–2 h after fogging, and ants were separated as part of arthropod ordinal sorting (see Dial et al., 2006 for results on ordinal arthropod assemblages). The ants sampled using fogging are mainly diurnal foraging species active during the sampling period (0700 to 0930), and therefore likely present a subset of the total local ant diversity.

All worker ants were identified as genus following Fayle et al. (2014), with relevant updates for taxonomy changes (Borowiec, 2016; Ward et al., 2015; Ward et al., 2016), and then separated into morphospecies. Where possible, species names were assigned using online image databases (www.antweb.org, www.antbase.net), published literature (Dorow & Kohout, 1995; Hung, 1970; Kohout, 2006a,b; Schödl, 1998), and the collections of TMF. Reproductive individuals were excluded from the data, since (i) their presence does not indicate an established colony, (ii) mating flights can confound estimates of abundance, and (iii) they can be challenging to match with workers unless entire nest series are collected. Lone major workers were also excluded for the latter reason. While all sample trays were suspended in the air at systematic, standardized horizontal and vertical positions, some trays captured no worker ants because there was only empty space with no foliage or stems between the sample tray without worker ants and the next sample tray above. In total, we obtained and identified ant assemblage samples for 61 out of 86 sampling points, with 14 samples having no ants, nine samples having been lost between sampling and analysis, and two samples in Transect 5 (two individual ants discovered) belonging to an emergent forest layer without horizontal positions for comparison.

#### 2.2.2 Microclimate and microhabitat connectivity

To quantify microclimate and habitat structure, air temperature (°C) and relative humidity (%) were measured at 0.5 h intervals over 24 h using Hobo Pro RH/Temperature Data Logger (Onset Computer Corporation). Data loggers were placed at 3 m intervals along each vertical transect, starting 1 m above the ground. At the data logger locations, photosynthetic photon flux density (PPFD) was recorded using a handheld light meter (Quantum Lightmeter, Spectrum Technologies, Plainfield, 1 L, USA) and normalized by dividing by maximum light value within each vertical transect to account for between‐day variation in lighting. The intent was to identify the relative (not absolute) light environment of the forest canopy (Dial et al., 2006). As microclimate was measured over a single 24‐h period for each transect, the dataset fails to capture variability at scales greater than a single day but reflects some spatial variation across the vertical plane sampled. Although we measured microclimate over a relatively short time, the daily variation and vertical pattern in air temperature and relative humidity are consistent with microclimate patterns from a nearby lowland primary forest site monitored over a longer period (128 consecutive days measure in Maliau Basin Conservation Area [4°49′N, 116°54′E], Hardwick et al., 2015).

We estimated one‐sided total leaf area between sampling trays as a measure of microhabitat connectivity at different sampling points for transects 1 to 6 (T1‐T6; Figure 2c). The leaf area within a sampling interval was calculated by multiplying the number of leaf intersections by the size of the base area of the interval, which was 1 m^2^ (the area of the sample tray). We then used these data to estimate leaf area index (LAI) over vertical intervals (sampling methods described in Dial, Bloodworth, et al., 2004; Dial et al., 2006, 2011; estimation methods in Dial et al., 2006; Dial et al., 2011). Conceptually, LAI refers to the number of leaf layers above the ground surface that would be pierced by a vertical line. For example, if LAI = 7, then there are, on average seven leaf layers above a random point on the ground within that height range; or 7 m^2^ of leaf area per m^2^ of the ground surface. We assumed (following MacArthur & Horn, 1969) that for any sample point in the canopy located at height *z* above the ground, the foliage density was approximately equal in all directions. Following this assumption at each height *z*, we systematically measured horizontal distances (*d*_*i*_) with a laser range finder to the nearest canopy element (foliage and stems) in 12 uniformly distributed azimuths every 2 m vertically from the ground to the height of the horizontal traverse line supporting the vertical transect. Using the *n* ≤ 12 distances to foliage at each sample point, we found the mean distance ($\bar{d}$) to foliage, doubled the mean (assuming that the observer was on the average midway between foliage elements), then inverted it to find leaf intersections per vertical meter at height *z* as LAI_z_ = 1/(2$\bar{d}$). By multiplying the LAI_z_ by collection area (1 m^2^) we estimated the leaf area sampled within the interval.

### 2.3 Data analyses

All statistical analyses were conducted in R version 4.0.0 (R Development Core Team, 2019).

#### 2.3.1 Assessing patterns in microclimate and microhabitat connectivity

We quantified microclimate (air temperature, relative humidity, and PPFD) and microhabitat connectivity (total leaf area) along both vertical and horizontal gradients. To be consistent with the assemblage data, we calculated the mean air temperature and relative humidity at the same scale (5 m vertical intervals) at which the ants were sampled by either using the data close to the 5 m interval point or using the average between sampling points. We expect microclimate and microhabitat connectivity to vary monotonically with vertical gradients due to canopy buffering effects, but nondirectionally along horizontal ones. As such, we ran linear regression models with air temperature, relative humidity, PPFD, and leaf area as response variables against vertical height above ground as a predictor covariate and horizontal position of vertical transects as a categorical predictor variable.

#### 2.3.2 Distribution of ant assemblages along vertical and horizontal gradients

To understand how ant richness and abundance changed vertically and horizontally, we used linear regression models. The abundance (*ln[x* + 1] transformed to control for heteroscedasticity) and richness of ants at each sampling point were used as response variables with vertical height as a continuous explanatory variable and horizontal position of vertical transects as a categorical explanatory variable.

To explore vertical and horizontal ant assemblage composition, we used nonmetric multidimensional scaling (NMDS) ordination of both abundance‐based (*ln*[*x* + 1] transformed) assemblages (Bray‐Curtis distances) and species presence/absence data (Jaccard distances) assemblages at each sampling point. Because results from ordinations using abundance and presence‐absence dissimilarities were similar, we mainly present abundance‐based ordination results. To increase sample size within groups for the vertical analyses, and so statistical power, we assigned all sampling points within 10 m bins to the same groups (i.e., 0–10 m, 10–20 m, etc.), testing for differences among these groupings using PERMANOVA (*adonis* function in the vegan package, 999 permutations; Oksanen et al., 2013, R Development Core Team, 2019).

To explore relationships between assemblage turnover and spatial distance, we conducted two different analyses. We calculated horizontal beta diversity by summing transect assemblage data across heights and vertical beta diversity by summing height assemblages across transects. We then calculated the pairwise assemblage dissimilarity across these summed data using the *beta.pair.abund* function (for abundance‐based dissimilarity) and *beta.pair* function (for presence‐absence data) in the betapart package (Baselga & Orme, 2012). We tested the effects of horizontal/vertical distance on pairwise assemblage dissimilarity between transects/strata by conducting multiple regressions on these distance matrices (*MRM* function in R ecodist package; Goslee & Urban, 2007). To test whether degree of turnover differed horizontally and vertically, we conducted replicated MRM analyses using individual transects to assess vertical turnover and individual heights to assess horizontal turnover. We then took the slopes and intercepts from the fitted MRMs and used linear models (function *lm* in R base package) to test for differences in the regression parameters horizontally and vertically. Slopes represent the strength of the distance‐decay relationship, with a more positive slope indicating a more rapid increase in dissimilarity. Intercepts represent the turnover at very small spatial scales (technically when distance = 0 m).

#### 2.3.3 Correlates of habitat factors and ant assemblage composition

We conducted constrained ordinations to understand how microclimate (air temperature relative humidity, and PPFD) and microhabitat connectivity (total leaf area) affect ant assemblage composition. We first conducted Detrended Correspondence Analysis (DCA) using the *decorana* function in the vegan package and found a maximum axis length greater than 4 (range: 3.91–6.96) indicating that canonical correspondence analysis (CCA) constrained ordinations that assume unimodal responses of species to environmental gradients were appropriate. We then checked the collinearity among all explanatory variables using *vif.cca* function to reduce redundancy in the model. No strong collinearity (VIF < 10) was detected between microclimate and microhabitat variables, and so all predictors were included in the Canonical Correspondence Analysis (CCA). We then conducted CCA ordinations using *cca* function in the vegan package with all explanatory variables (air temperature, relative humidity, PPFD, and total leaf area). We conducted backward model selection using the *ordistep* function in the vegan package to identify the most significant variables affecting the assemblage composition based on permutation tests using 1000 permutations (Blanchet et al., 2008). As the model selection process for CCA analysis requires samples with all environmental factors available, only 48 out of total 61 ant assemblages that had all environmental information available were included for this analysis.

## 3 RESULTS

### 3.1 Patterns of microclimate and microhabitat connectivity

**FIGURE 3:**
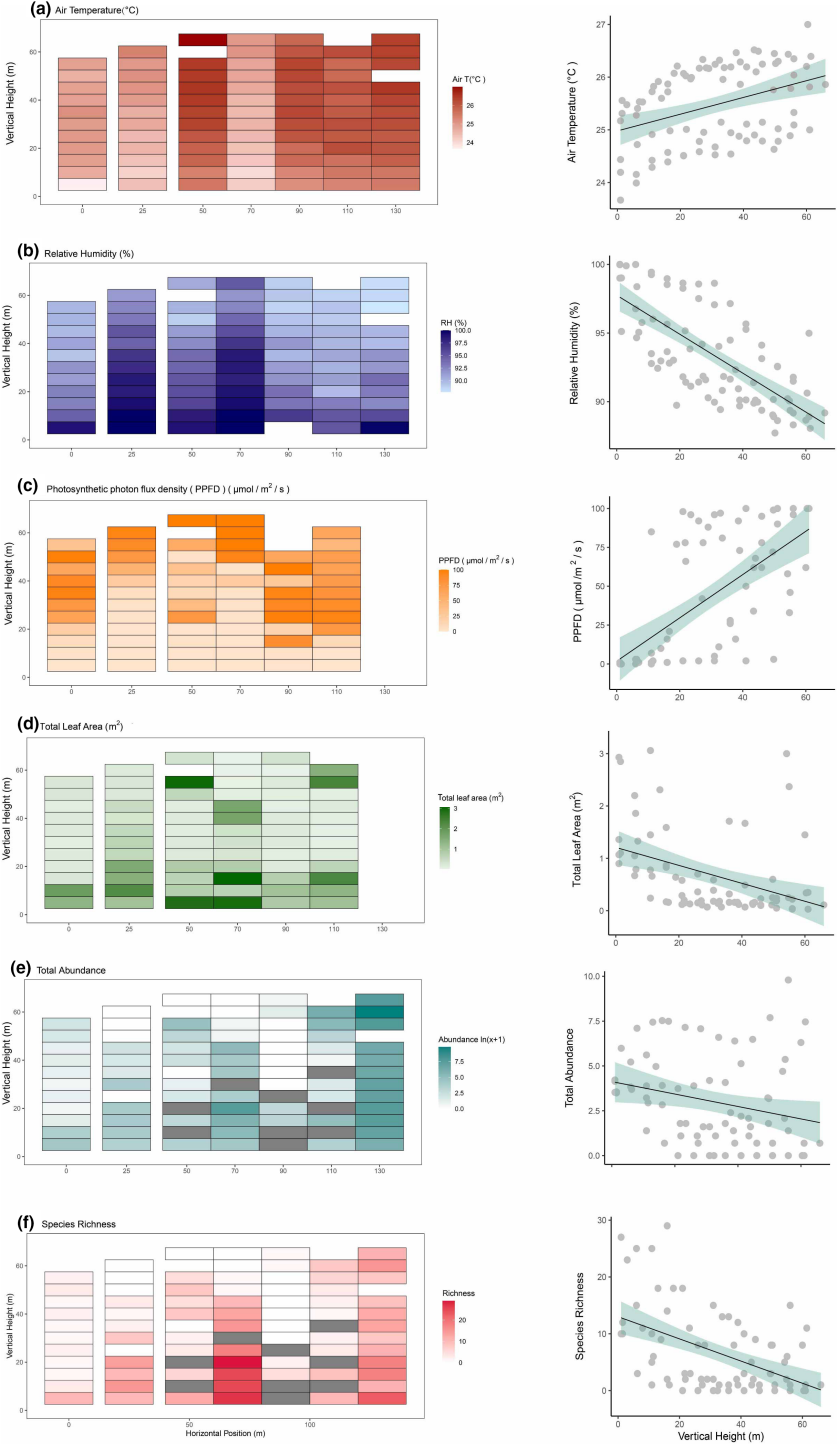
Heatmaps and scatter plots showing the horizontal and vertical distributions of (a) air temperature, (b) relative humidity, (c) photosynthetic photon flux density (PPFD), (d) total leaf area, (e) ant species abundance (*ln*[*x* + 1] transformed)), and (f) ant species richness. Colors with higher saturation indicate higher values of the variables. Plots with missing ant samples were filled with gray color in (e) and (f).

We found high horizontal and vertical variation in microclimate and microhabitat connectivity in our study site (Figure 3; air temperature: *R* ^2^ = .91, *F*_7,78_ = 106.90, *p* < .001; relative humidity: *R* ^2^ = .89, *F*_7,77_ = 90.17, *p* < .001). In 10 m intervals from the forest floor, temperature increased by 0.13°C and relative humidity decreased by 1.4% (Figure 3; Table 2). In addition, there was nondirectional variation between horizontal locations in temperature (0.36°C per 10 m) and humidity (1.4% per 10 m; Table 2). Photosynthetic photon flux density (PPFD) increased with vertical height (as PPFD was normalized within each vertical transect, we could not compare this across transects, note that PPFD data was missing for Transect 7; *R* ^2^ = .39, *F*_1,68_ = 43.34, *p* < .001; Table 2, Figure 3). Total leaf area generally decreased towards the upper canopy but did not show significant horizontal variation (note that leaf area data were missing for Transect 7; *R* ^2^ = .24, *F*_6,67_ = 3.53, *p* = .004; Table 2, Figure 3).

**TABLE 2:** Effects of vertical and horizontal (Hrzt) position on air temperature and relative humidity based on linear regression analyses. Coefficients with statistical significance shown in boldface. Significant *p* values are labeled with *

Response	Model factor	Coefficient	Std. Error	*t* value	Pr(>|*t*|)
Air temperature	Vertical height (m)	**0.013**	0.001	9.951	<.001*
	Hrzt 0 m (intercept)	24.404	0.081	297.740	<.001*
	Hrzt 25 m	−0.044	0.100	−0.441	.661
	Hrzt 50 m	**1.358**	0.100	13.572	<.001*
	Hrzt 70 m	**−0.290**	0.096	−3.005	.004*
	Hrzt 90 m	**1.058**	0.097	10.926	<.001*
	Hrzt 110 m	**1.032**	0.100	10.312	<.001*
	Hrzt 130 m	**1.247**	0.100	12.451	<.001*
Relative humidity	Vertical height (m)	**−0.137**	0.007	−18.304	<.001*
	Hrzt 0 m (intercept)	95.867	0.430	223.009	<.001*
	Hrzt 25 m	**4.523**	0.522	8.663	<.001*
	Hrzt 50 m	**2.084**	0.522	3.989	<.001*
	Hrzt 70 m	**5.301**	0.513	10.334	<.001*
	Hrzt 90 m	0.144	0.516	0.281	.779
	Hrzt 110 m	**−0.559**	0.522	−1.071	.288
	Hrzt 130 m	0.294	0.523	0.563	.575
Photosynthetic photon flux density (PPFD)	Vertical height (m)	**1.392**	0.212	6.584	<.001*
Total leaf area	Vertical height (m)	**−0.017**	0.005	−3.684	<.001*
	Hrzt 0 m (intercept)	0.910	0.254	3.572	<.001*
	Hrzt 25 m	0.381	0.305	1.251	.215
	Hrzt 50 m	0.432	0.305	1.414	.162
	Hrzt 70 m	**0.551**	0.300	1.839	.070
	Hrzt 90 m	−0.029	0.296	−0.097	.923
	Hrzt 110 m	0.443	0.306	1.451	.151

### 3.2 Distribution of ant assemblages along vertical and horizontal gradients

**FIGURE 4:**
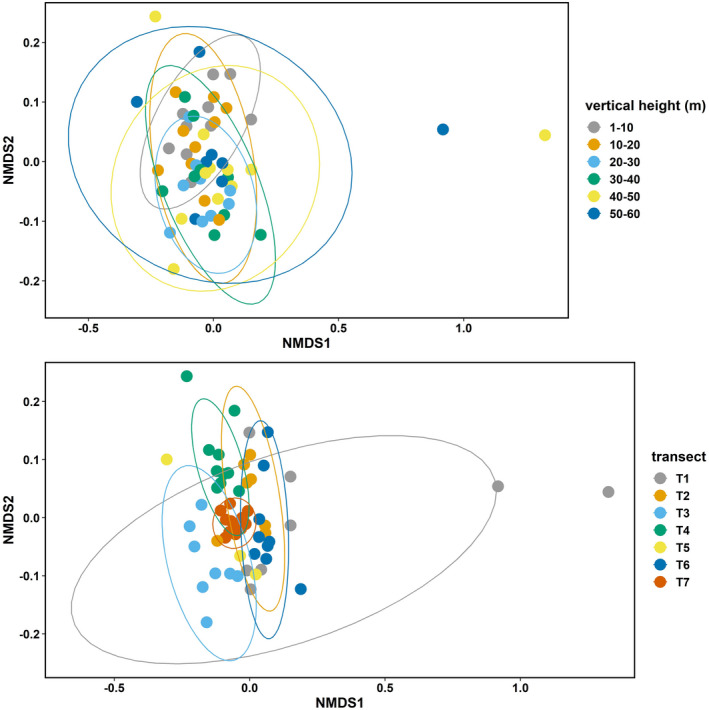
NMDS plot of abundance‐based (*ln*[*X* + 1]transformed) ant communities grouped by vertical strata and transect (horizontal position). Transects are shown by color, and ellipses show the 95% confidence intervals around the centroids of each transect.

**FIGURE 5:**
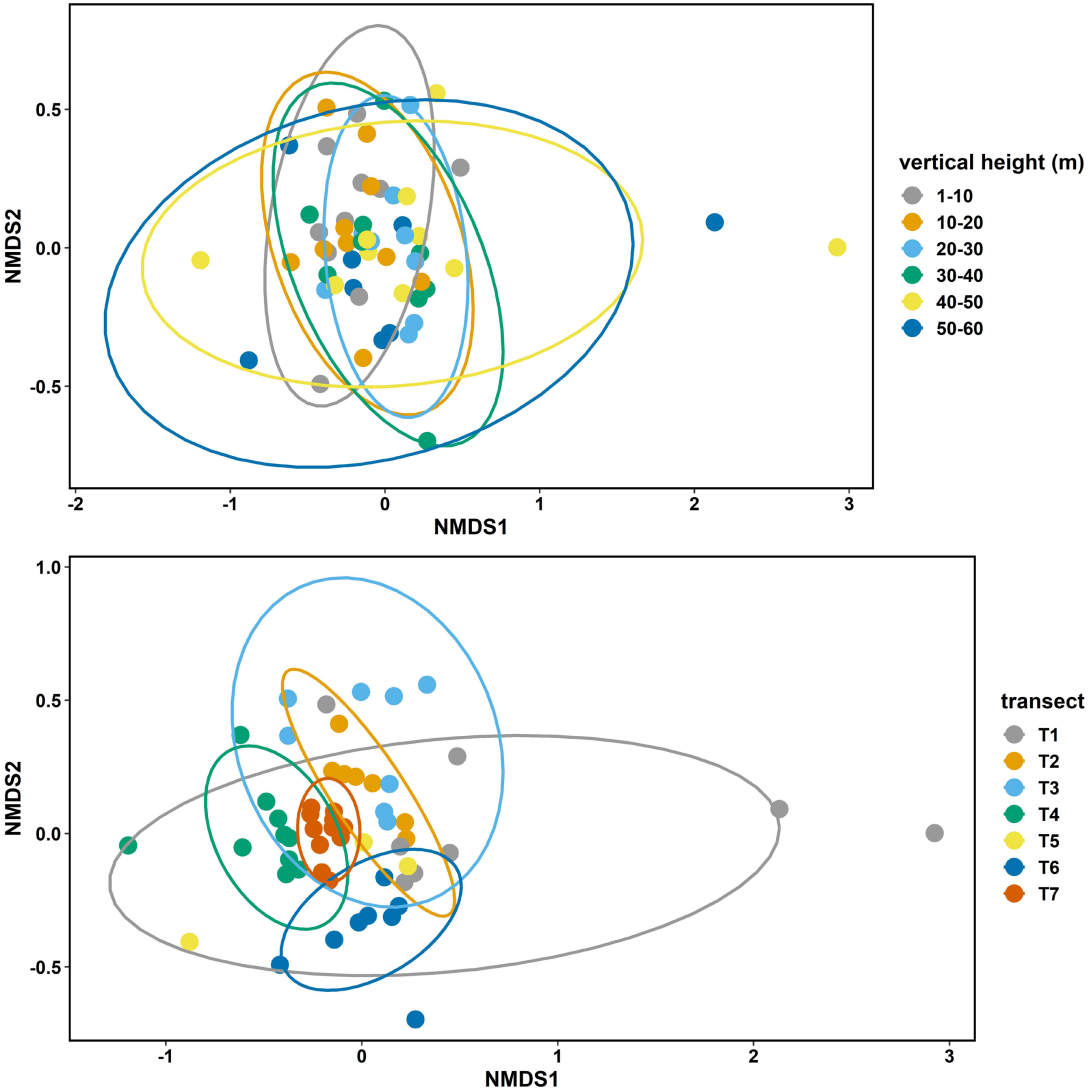
NMDS plot of presence‐absence‐based ant assemblages grouped by vertical strata and transect (horizontal position). Transects are shown by color, and ellipses show the 95% confidence intervals around the centroids of each transect.

In total, 35,710 individual ants from 138 species in 31 genera were sampled. Ant abundance decreased significantly with increasing height in the canopy from an average of 395 individuals per m^2^ at 5 m, to 101 individuals per m^2^ at 60 m (*ln*[*x* + 1] tranformed, *R* ^2^ = .67, *F*_7,59_ = 16.68, *p* < .001). Ant species richness also significantly decreased with height in the canopy, from an average of 12 species per m^2^ at 5 m to an average of 3 species per m^2^ at 60 m (*R* ^2^ = .66, *F*_7,59_ = 16.65, *p* < .001). Both ant abundance and richness also significantly varied across transects (horizontal positions; Table 3, Figure 3). Ant community assemblages showed significant differences in species composition both vertically and horizontally (Figures 4 and 5; PERMANOVA analysis, vertical stratification: *F* = 1.8, *p* = .01, *R* ^2^ = .09; across transects: *F* = 6.4, *p* = .001, *R* ^2^ = .39).

**TABLE 3:** Effects of vertical and horizontal position on species abundance (*ln*[*X* + 1]transformed) and richness based on linear regresssion analyses. Coefficients with statistical significance shown in boldface. Significant *p* values are labeled with *

Response	Model factor	Coefficient	Std. Error	*t* value	Pr(>|*t*|)
Total abundance (*ln*[*x* + 1] transformed)	Vertical height (m)	**−0.023**	0.010	−2.277	.026*
	Hrzt 0 m (intercept)	2.381	0.527	4.516	<.001*
	Hrzt 25 m	0.326	0.620	0.525	.602
	Hrzt 50 m	1.036	0.691	1.499	.139
	Hrzt 70 m	**2.317**	0.650	3.567	<.001*
	Hrzt 90 m	0.345	0.815	0.424	.673
	Hrzt 110 m	**1.657**	0.672	2.466	.017*
	Hrzt 130 m	**5.563**	0.621	8.950	<.001*
Species richness	Vertical height (m)	**−0.167**	0.030	−5.511	<.001*
	Hrzt 0 m (intercept)	7.068	1.590	4.446	<.001*
	Hrzt 25 m	2.943	1.872	1.572	.121
	Hrzt 50 m	**4.212**	2.085	2.020	.048*
	Hrzt 70 m	**13.675**	1.959	6.979	<.001*
	Hrzt 90 m	1.948	2.457	0.793	.431
	Hrzt 110 m	3.677	2.028	1.813	.075
	Hrzt 130 m	**11.286**	1.875	6.019	<.001*

**FIGURE 6:**
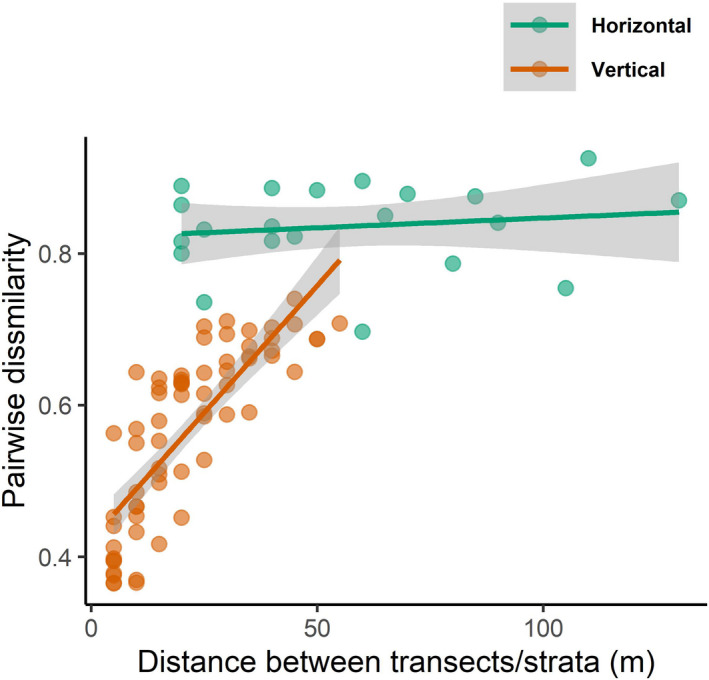
Scatter plot showing the effects of horizontal and vertical distance on abundance‐based pairwise dissimilarity of ant assemblages. Gray bands show 95% confidence intervals. Dissimilarity close to one indicates that two assemblages are highly different, while dissimilarity close to zero indicates that they are very similar.

We pooled data within the same transect or vertical stratum to examine distance‐decay patterns between transects/strata. This showed strong effects of vertical distance on pairwise dissimilarity indexes between vertical strata (MRM analyses: coefficient = 0.007, *p* = .001, *R* ^2^ = .65, Figure 6). In contrast, we found no effects of horizontal distance on pairwise assemblage dissimilarity between transects (MRM analyses: coefficient = 0.0003, *p* = .52, *R* ^2^ = .02, Figure 6). The pairwise dissimilarity was consistently higher horizontally than vertically (Figure 6).

**FIGURE 7:**
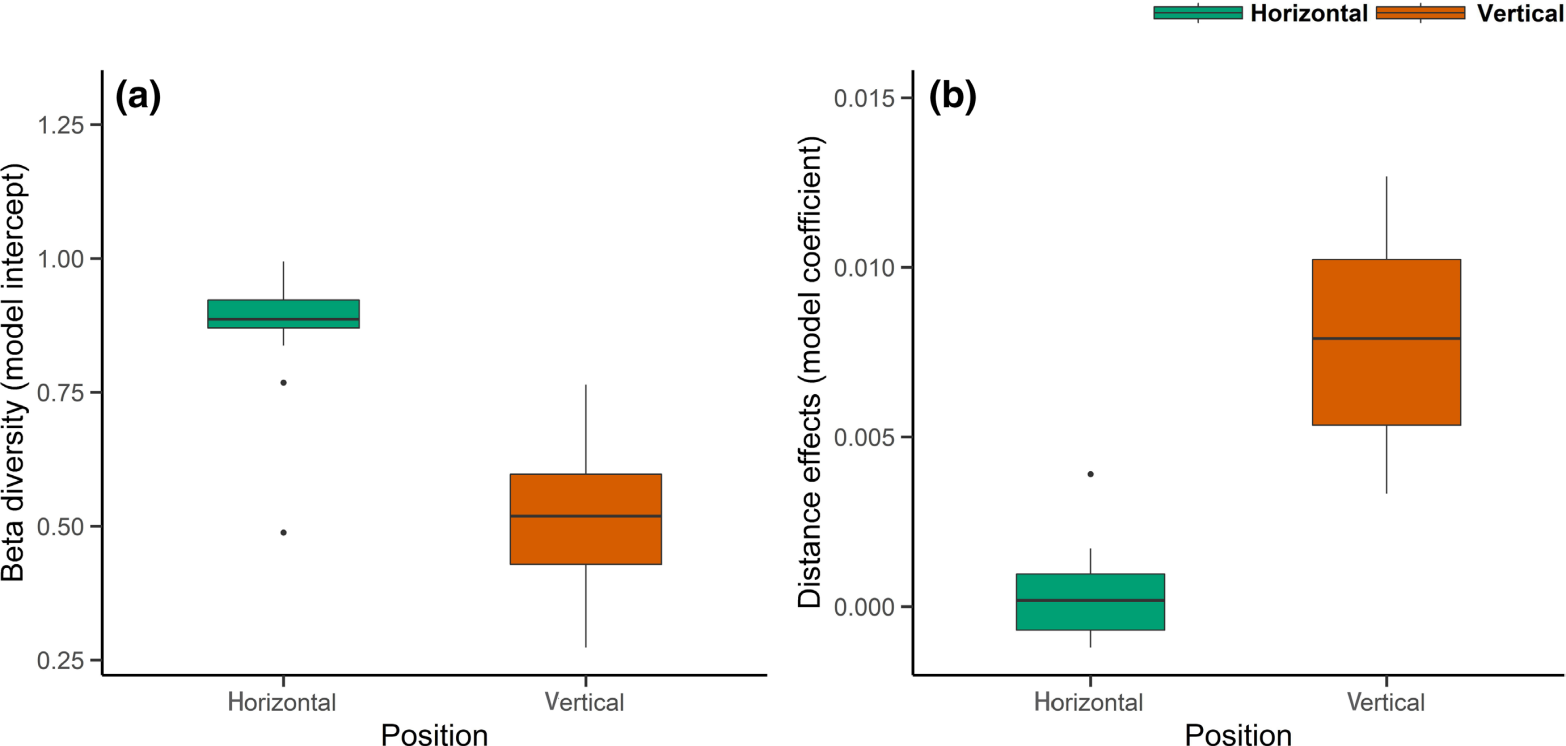
Boxplots showing the median and interquartile ranges (IQR) of the estimated very small‐scale beta diversity (model intercept) and distance effects (model coefficient) on pairwise dissimilarity between sampling points within transects/strata. Model intercepts and coefficients are from the linear regressions between distance and assemblage dissimilarity within each horizontal transect and vertical stratum (see Figures 8 and 9). Whiskers denote 1.5 * IQR. Data beyond this range are plotted individually.

**FIGURE 8:**
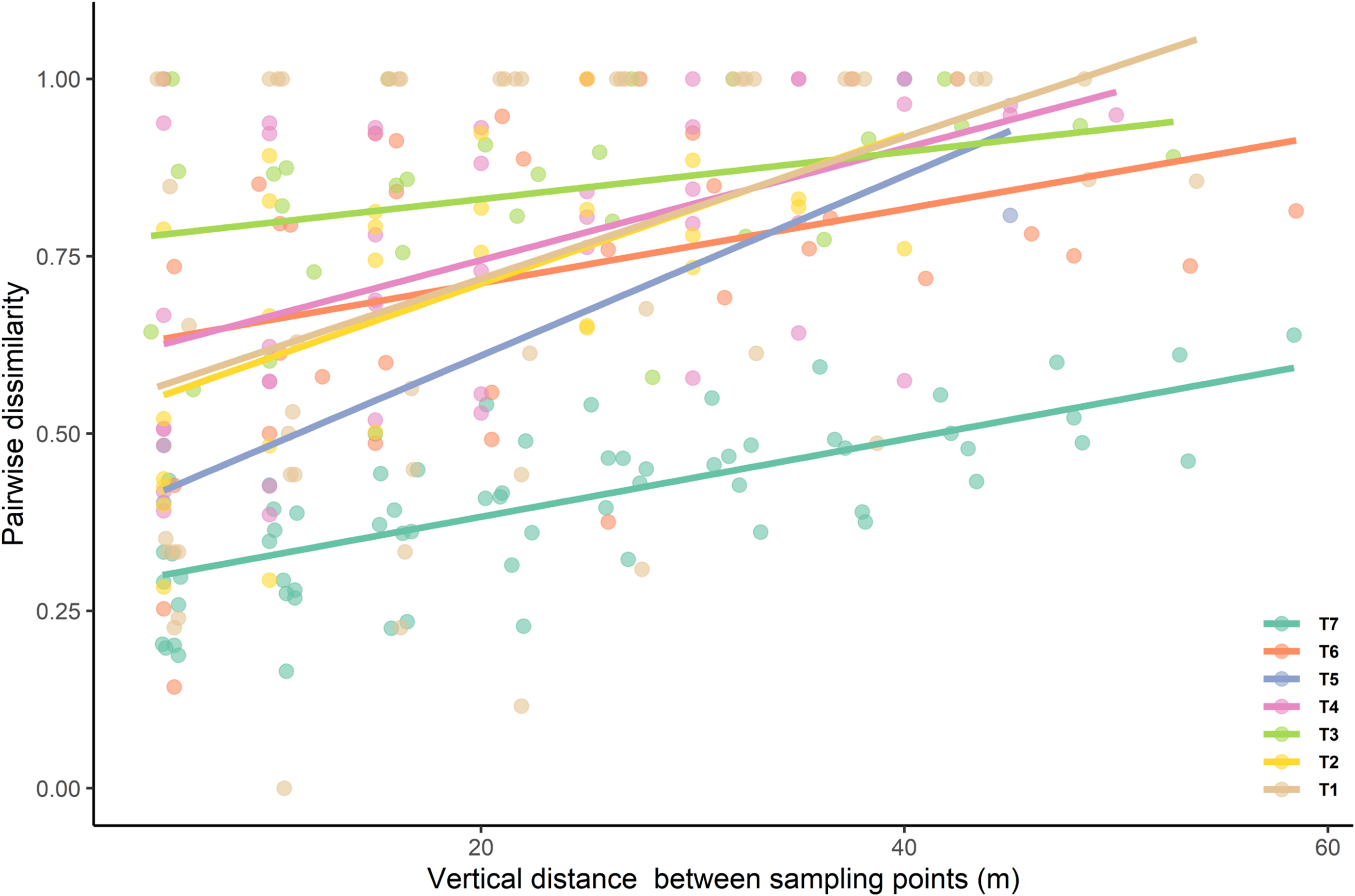
Scatter plots showing the distance‐decay patterns of ant assemblages (abundance‐based) within each vertical transect. Pairwise dissimilarities from different vertical transects are shown by color.

**FIGURE 9:**
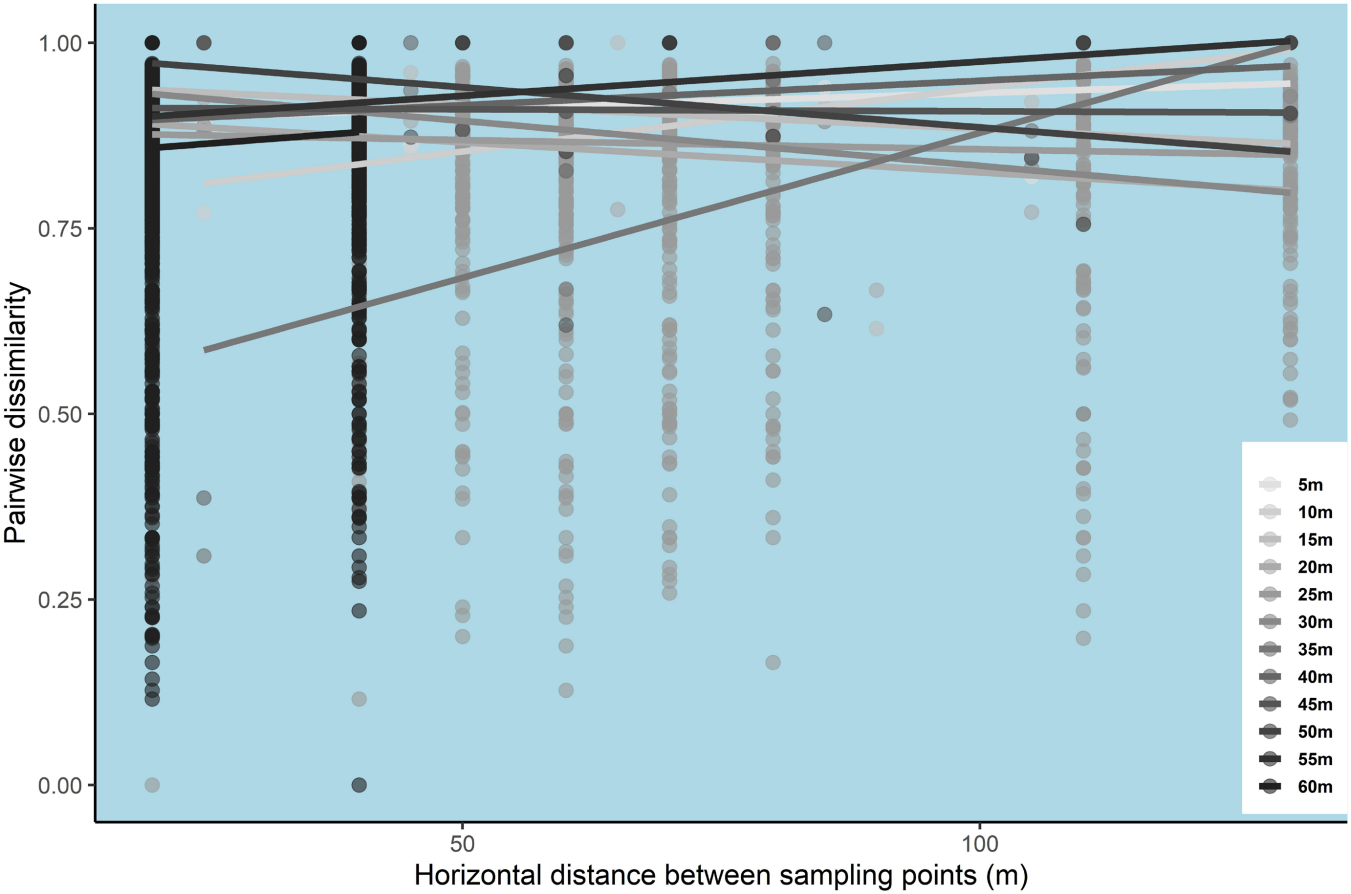
Scatter plot showing the distance‐decay patterns of ant assemblages (abundance‐based) within each horizontal stratum. Pairwise dissimilarities from different vertical transects are shown in a different color along a gray color gradient. Colors closer to black indicate vertical positions closer to the canopy, and colors closer to white indicate vertical positions closer to the ground.

By comparing the effects of vertical distance on assemblage dissimilarity within vertical transect and the effects of horizontal distance within vertical strata, we found greater small‐scale pairwise dissimilarity (higher intercepts) over horizontal distance within the same vertical strata than over vertical distance within the same vertical transect (linear regression: *F*_1,17_ = 25.93, *p* < .001, *R* ^2^ = .60, Table 4, Figure 7a). However, the effects of vertical distance on pairwise dissimilarity were much stronger than that of horizontal distance, presenting a vertical distance‐decay pattern within each transect (linear regression: *F*_1,17_ = 46.45, *p* < .001, *R* ^2^ = .73, Tables 4 and 5, Figures 7b, 8 and 9).

**TABLE 4:** Effects of vertical and horizontal distance on pairwise dissimilarity of abundance‐based ant assemblages within each transect and vertical stratum based on results from Multiple Regression on Distance Matrices (MRM) analysis. Coefficient value with statistical significance shown in boldface

Transect	Intercept	Coefficient
T1	0.519	**0.010**
T2	0.502	**0.010**
T3	0.764	0.003
T4	0.586	**0.008**
T5	0.356	0.013
T6	0.608	0.005
T7	0.273	**0.005**

Vertical stratum	Intercept	Coefficient
H1	0.89	4.236e‐4
H2	0.768	0.002
H3	0.950	−6.573e‐4
H4	0.907	−8.112e‐4
H5	0.882	−2.490e‐4
H6	0.955	−0.001
H7	0.488	0.004
H8	0.883	6.590e‐4
H9	0.913	−5.485e‐5
H10	0.994	−0.001
H11	0.883	0.001
H12	0.837	0.001

**TABLE 5:** Effects of vertical and horizontal distance on presence‐absence‐based pairwise dissimilarity of ant assemblages within each transect and vertical stratum based on results from Multiple Regression on distance Matrices (MRM) analysis. Coefficient values with statistical significance shown in boldface. Significant *p* values are labeled with *

Transect	Intercept	Coefficient
T1	0.517	**0.01**
T2	0.601	**0.009**
T3	0.827	0.002
T4	0.679	**0.006**
T5	0.841	0.001
T6	0.576	**0.007**
T7	0.503	**0.006**

Vertical stratum	Intercept	Coefficient
H1	0.906	0.000
H2	0.841	0.001
H3	0.947	0.000
H4	0.966	−0.001
H5	0.944	−0.001
H6	0.969	−0.001
H7	0.641	0.003
H8	0.901	0.001
H9	0.927	0.000
H10	0.992	−0.001
H11	0.921	0.001
H12	0.813	0.005

### 3.3 Correlates of ant assemblage composition

**FIGURE 10:**
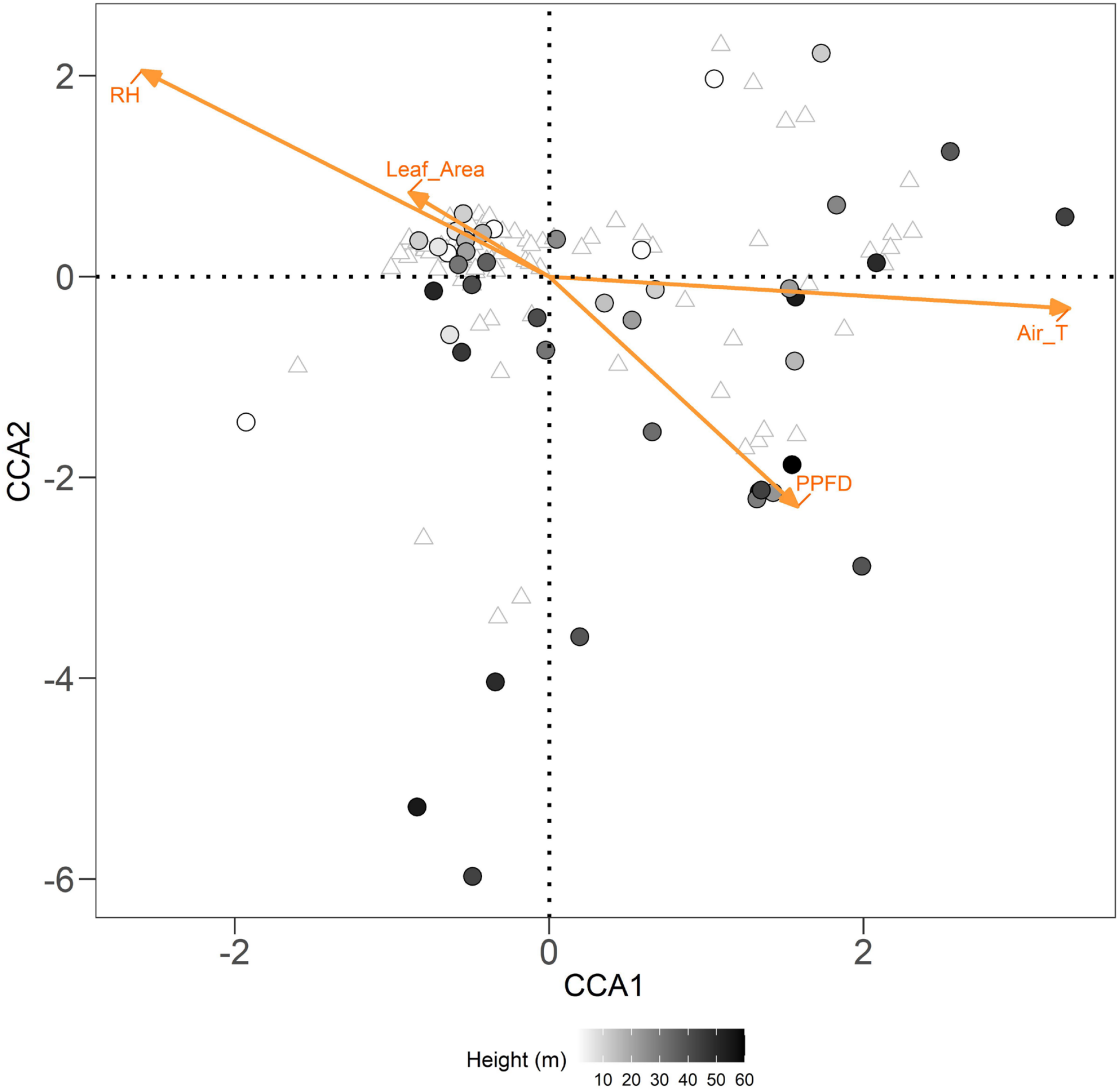
Canonical correspondence analysis (CCA) of ant community composition explained by effects of microclimate (air temperature, relative humidity, and photosynthetic photon flux density (PPFD)) and vegetation profile (total leaf area). Circular points represent different sampling locations. Darker colors show a higher vertical position. Triangles represent ant species.

Results from canonical correspondence analysis (CCA) backward model selection indicated that both air temperature and relative humidity were significantly associated with ant assemblage composition, while PPFD and total leaf area were not (Figure 10, Table 6).

**TABLE 6:** Backward model selection result for canonical‐correlation analysis (CCA). Significant *p* values are labeled with *

Full model (ant assemblage composition ~ Total leaf area + PPFD + air temperature + relative humidity)				
Explanatory factors	*df*	AIC	*F*	*p* value
Total leaf area	1	225.51	0.948	.530
PPFD	1	225.49	0.929	.505
Relative humidity	1	226.36	1.735	.010*
Air temperature	1	227.33	2.652	.005*

Model without “Total leaf area” (ant assemblage composition ~ PPFD + air temperature + relative humidity)				
Explanatory factors	*df*	AIC	*F*	*p* value
PPFD	1	224.49	0.911	.505
Relative humidity	1	225.37	1.741	.010*
Air temperature	1	226.30	2.641	.005*

Model without “Total leaf area” and “PPFD” (ant assemblage composition ~ air temperature + relative humidity)				
Explanatory factors	*df*	AIC	*F*	*p* value
Relative humidity	1	225.25	2.660	.005*
Air temperature	1	225.35	2.759	.005*

## 4 DISCUSSION

We observed different patterns in species assemblage distribution over small‐scale (tens of metres) vertical and horizontal gradients in a complex habitat. The high local diversity of arboreal ants can be explained by high environmental and habitat heterogeneity along both horizontal and vertical dimensions. We found consistently greater horizontal turnover in ant assemblages than that observed at comparable vertical distances. However, a distance‐decay pattern of ant turnover was only detected vertically, but not horizontally, with the high species turnover between horizontal positions being independent of distance. The observed patterns confirmed our hypotheses that turnover is likely associated with continuous directional changes in microclimate vertically and stochastic environmental variation horizontally (Figure 1a). The higher horizontal assemblage turnover than vertical turnover at short distances is likely due to low microhabitat connectivity horizontally (Figure 1b).

We found the spatial distribution of ant assemblages was associated with heterogeneity in air temperature and relative humidity within the three‐dimensional structure of the forest. The spatial pattern of microclimate in our study plot agrees with other studies in the tropical rainforest: air temperature increased with height above ground, while relative humidity decreased (Davis et al., 2019; De Frenne et al., 2019). Horizontal variation in microclimate was also high but without directional change, presumably relating to variation in vegetation structure within and between trees (Fetcher et al., 1985; Scheffers et al., 2017), which may explain the high turnover of ant assemblages and lack of distance‐decay pattern in this dimension at the scales we sampled. Tropical arboreal ants show thermal adaptation to their vertical habitat use through their physiology (Kaspari et al., 2015), morphology (Law et al., 2020), and nesting site selection (Plowman et al., 2019). The high variance in microclimate generates diverse thermal niches for ant species with different thermal tolerances and hence can facilitate the coexistence of multiple species at small spatial scales (Lessard et al., 2009). For tiny ectotherms like ants, fine‐scale environmental heterogeneity can play an important role in defining their distributions, probably due to the small foraging range and small body size of ants, and the thermal diversity of the environment (Bütikofer et al., 2020; Fayle et al., 2010; Kaspari et al., 2015; Klimes et al., 2012; Ribas & Schoereder, 2007). Indeed, the fact that there was a distance‐decay pattern with vertical height despite the presumably better connectivity in this dimension (see below) indicates a strong impact of environmental filtering. The association between microclimate pattern and ant assemblage composition may result not only from direct effects through environmental preferences of ant species, but can also be a consequence of indirect biotic effects, for example, if microclimate influences ant food availability. However, the relative importance of direct and indirect microclimate influences on ant assemblage composition remains to be investigated.

In addition to microclimate, biotic influences such as resource limitation and vegetation structure may also contribute to the high horizontal turnover we observed. The decline in ant richness and abundance with height in the canopy could be due to reduction in leaf area, which limits foraging range and nest site availability (Adams et al., 2019; Plowman et al., 2019). Ant assemblage composition can also be affected by vegetation structure such as tree size, number of branches, and cavity diversity (Adams et al., 2019; Plowman et al., 2019; Powell et al., 2011; Yusah & Foster, 2016). Hence, we can expect distinct ant communities to be hosted by different individual trees and that the distance between trees per se may not drive assemblage dissimilarity alone. The lack of distance effects on high horizontal turnover arboreal ants in our study is consistent with patterns observed in the canopies of other forests for canopy ant assemblages across greater horizontal distances (100–700 m), whereas a distance‐decay pattern was observed in ground ant assemblages (Antoniazzi et al., 2021). For social insects like ants where workers are wingless, vertical movement of workers within the colony tree is likely to be less challenging than movement between trees, especially without vegetation connections such as lianas (Adams et al., 2019; Yusah & Foster, 2016), which may be less common in the Asian tropics compared with the American tropics (Dial, Bloodworth, et al., 2004). Such a lack of connectivity between individual trees means that each tree canopy might function as an island within the forest (Adams et al., 2017; Southwood & Kennedy, 1983).

Our findings offer important insights into the way in which biodiversity is maintained at fine spatial scales in complex three‐dimensional habitats. The high species richness discovered within the 130 m long by 70 m high vertical plot in our study site represents a high proportion of ant diversity at larger scales. The number of species that we detected using precision fogging across vertical strata among 11 trees reached about 40% of the number of species sampled in a similar forest habitat elsewhere in Sabah from 99 trees (Floren et al., 2014). This finding of a relatively large proportion of regional biodiversity being sampled from small plots within rainforests is in line with patterns for herbivorous insects (Novotny et al., 2007), birds (Huang & Catterall, 2021), and butterflies (Daily & Ehrlich, 1995) and is likely driven by high structural complexity at small scales (Basset et al., 2012).

Our findings must be interpreted within the limits of short sampling time and small sample size. Microclimate information was only monitored for 24 h for each transect, although this is likely representative of longer‐term patterns (see above). Future studies involving long‐term monitoring of microclimate are needed to understand the distribution of microclimate and how it may affect assemblage distribution within the complex three‐dimensional structure of tropical rainforests. In addition, although our sampling intensity per tree was high, we sampled across a relatively limited spatial area and some samples were missing. Future research should expand on our work by increasing the number of replicates and the spatial scale.

## 5 CONCLUSION

Our study revealed high spatial variation in ant communities in both horizontal and vertical dimensions over small spatial scales. We detected a distance‐decay pattern in ant assemblage composition vertically, but no effects of distance on assemblage turnover horizontally. These patterns are associated with vertical variation in air temperature and relative humidity. We found higher horizontal than the vertical turnover in ant assemblages, which may be driven by the low microhabitat connectivity. Our findings demonstrate the need to consider species turnover in both spatial dimensions. Such insights will enable us to understand the mechanisms behind species distributions, and therefore make more informed decisions about how to preserve biodiversity in these highly diverse tropical forests, which are currently threatened by anthropogenic change.

## AUTHOR CONTRIBUTIONS

**Shuang Xing:** Conceptualization (lead); formal analysis (lead); writing – original draft (lead). **Amelia Hood:** Investigation (equal); writing – review and editing (equal). **Roman Dial:** Investigation (equal); methodology (equal); writing – review and editing (equal). **Tom Fayle:** Conceptualization (equal); formal analysis (equal); methodology (equal); supervision (supporting); writing – review and editing (equal).

## CONFLICT OF INTEREST

There is no conflict of interest among authors.

## Supporting information


Data S1

## Data Availability

Data are available at Dryad: https://doi.org/10.5061/dryad.12jm63z1g.
