# Supplementary material for: Species turnover in ant assemblages is greater horizontally than vertically in the world's tallest tropical forest
Source: Ecol Evol. 2022 Jul 29;12(8):e9158. doi: 10.1002/ece3.9158 (PMC9336171; doi:10.1002/ece3.9158)
Supplement: Supplementary file 1 — Data S1 [file ECE3-12-e9158-s001.docx]

**Supplementary data.**

Ant abundance and species richness at each sampling point

| Horizontal position | Vertical hight (m) | Ant abundance | Species richness |
| --- | --- | --- | --- |
| 0 | 1 | 67 | 10 |
| 0 | 49.7 | 5 | 3 |
| 0 | 54.8 | 7 | 2 |
| 0 | 6.3 | 39 | 3 |
| 0 | 11 | 3 | 1 |
| 0 | 16.7 | 1 | 1 |
| 0 | 21.9 | 2 | 2 |
| 0 | 27.4 | 1 | 1 |
| 0 | 32.9 | 2 | 1 |
| 0 | 38.6 | 3 | 2 |
| 0 | 43.5 | 2 | 1 |
| 25 | 1 | 33 | 10 |
| 25 | 46 | 0 | 0 |
| 25 | 51 | 0 | 0 |
| 25 | 56 | 0 | 0 |
| 25 | 6 | 45 | 15 |
| 25 | 11 | 48 | 10 |
| 25 | 16 | 49 | 14 |
| 25 | 21 | 0 | 0 |
| 25 | 26 | 48 | 8 |
| 25 | 31 | 4 | 2 |
| 25 | 36 | 5 | 1 |
| 25 | 41 | 8 | 3 |
| 50 | 1.5 | 32 | 12 |
| 50 | 49.8 | 23 | 8 |
| 50 | 54.2 | 109 | 5 |
| 50 | 11.5 | 18 | 6 |
| 50 | 21.7 | 5 | 3 |
| 50 | 27.1 | 2 | 2 |
| 50 | 37.7 | 10 | 8 |
| 50 | 43.4 | 7 | 5 |
| 70 | 1 | 62 | 27 |
| 70 | 46 | 1 | 1 |
| 70 | 51 | 1 | 1 |
| 70 | 6 | 184 | 25 |
| 70 | 11 | 273 | 25 |
| 70 | 16 | 1862 | 29 |
| 70 | 21 | 41 | 18 |
| 70 | 31 | 65 | 15 |
| 70 | 36 | 58 | 13 |
| 70 | 41 | 168 | 10 |
| 90 | 56 | 3 | 1 |
| 90 | 61 | 1 | 1 |
| 90 | 66 | 1 | 1 |
| 90 | 11 | 24 | 5 |
| 90 | 16 | 16 | 2 |
| 110 | 1.5 | 34 | 10 |
| 110 | 49.5 | 24 | 3 |
| 110 | 55 | 213 | 6 |
| 110 | 60 | 548 | 8 |
| 110 | 14 | 143 | 9 |
| 110 | 23.5 | 5 | 3 |
| 110 | 29 | 4 | 2 |
| 110 | 39 | 1 | 1 |
| 110 | 44 | 4 | 2 |
| 130 | 3 | 397 | 23 |
| 130 | 50.2 | 2194 | 8 |
| 130 | 56 | 17921 | 15 |
| 130 | 61.4 | 1733 | 11 |
| 130 | 8 | 1213 | 11 |
| 130 | 13 | 1709 | 18 |
| 130 | 18 | 1789 | 18 |
| 130 | 23.25 | 1277 | 11 |
| 130 | 28.2 | 1182 | 8 |
| 130 | 33.9 | 731 | 13 |
| 130 | 39 | 596 | 12 |
| 130 | 44.7 | 654 | 9 |
